# Google Trends for the Human Papillomavirus Vaccine in India From 2010 to 2024: Infodemiological Study

**DOI:** 10.2196/69729

**Published:** 2025-05-27

**Authors:** Rashmi Mehra, Arindam Ray, Amrita Kumari, Amanjot Kaur, Rhythm Hora, Syed F Quadri, Seema Singh Koshal, Bodhisatwa Ray, Shyam Kumar Singh, Abida Sultana, Arup Deb Roy

**Affiliations:** 1 John Snow India, Plot No. 5 & 6, First Floor Allied House, Pocket 10, Sector B, Vasant Kunj, New Delhi, 110070, India, 91 9560213460; 2 Bill and Melinda Gates Foundation, New Delhi, India; 3 University of Exeter, Exeter, United Kingdom

**Keywords:** HPV, cervical cancer, HPV vaccine, Google Trends, public health, India, human papillomavirus

## Abstract

**Background:**

Human papillomavirus (HPV) is a leading cause of cervical cancer. It has a substantial impact on global public health, with low- and middle-income countries, including India, facing the highest burden. In 2022, India reported 127,526 new cases and 79,906 deaths due to cervical cancer, projected to increase by 61% by 2040. Although the National Technical Advisory Group on Immunization recommended the HPV vaccine for cervical cancer prevention, it is yet to be a part of India’s universal immunization program.

**Objective:**

This study aims to examine online interest in the HPV vaccine in India from January 2010 to April 2024 using Google Trends.

**Methods:**

A cross-sectional analysis of Google Trends data was performed, using the relative search volume to track interest on a scale of 0‐100. Trends were analyzed annually using 1-way ANOVA and joinpoint regression to identify significant changes in search behavior related to public health events. Statistical significance was set at *P*<.05.

**Results:**

The average annual growth in HPV vaccine-related searches was 13.7% (95% CI 7.9%‐19.1%), with the highest relative search volume in 2024 (49.5) and the lowest in 2017 (3.38). Spikes in search interest aligned with key events like the 2018 National Technical Advisory Group on Immunization recommendation and the 2022 launch of the indigenous HPV vaccine. The results highlight online search data’s value in tracking public interest, which fluctuates in response to health policy changes or developments on social media. In India, targeted digital strategies will be vital for addressing vaccine hesitancy and increasing HPV vaccine uptake.

**Conclusions:**

Google Trends data can inform public health strategies by identifying periods of high interest, aiding in the promotion of HPV vaccination in India.

## Introduction

### Background

Human papillomavirus (HPV) is recognized as one of the leading causes of cervical cancer, contributing to substantial global morbidity and mortality rates in females [1]. In 2022, cervical cancer accounted for approximately 660,000 new cases and over 350,000 deaths worldwide, with low- and middle-income countries bearing the highest burden. In India, the situation is alarming, with 127,526 new cases and 79,906 deaths attributed to cervical cancer in 2022 [2]. The mortalities due to cervical cancer in India in 2040 are estimated to be 124,677—an increase of 61% over the estimated number of deaths due to cervical cancer in 2020 [3]. Cervical cancer, largely preventable through vaccination against HPV, remains a pressing public health concern [4]. HPV vaccines are recommended for adolescent girls to prevent cervical cancer and other HPV-related diseases [5]. In India, in the year 2017, the National Technical Advisory Group on Immunization endorsed the vaccine for adolescent girls, emphasizing its importance in public health strategy against cervical cancer [6]. However, HPV vaccination has not yet been incorporated into India’s universal immunization program, contributing to a substantial gap in protection against cervical cancer. This omission possibly culminates into broader challenges, including limited public interest, awareness, and accessibility that could eventually influence vaccine acceptance [7].

In today’s digital era, Google searches are a widely used method for finding information online, and the HPV vaccine has ranked among the most frequently discussed topics on the internet worldwide [8-10]. Research across various social media platforms, including YouTube [11], Facebook [12], Instagram [13], and Twitter [14,15], indicates that a significant amount of HPV vaccine-related misinformation can contribute to negative public perceptions of the vaccine [16].

Google Trends (GT) is a widely used tool for analyzing online search behaviors and search patterns in health care and public health research [17]. It allows for tracking changes in online interest in specific terms across various countries or regions over selected periods and enables comparisons between regions [18]. Data from GT have been valuable for monitoring trends in health information-seeking behavior, often aiding in predicting or detecting outbreaks [19-23]. The field of “infodemiology” focuses on studying these online behaviors by analyzing internet data, including GT, and is defined as “the science of distribution and determinants of information in an electronic medium, specifically the Internet, or in a population, with the goal of informing public health and policy” [24].

While multiple studies have investigated HPV vaccine-related misinformation [10-13] and vaccine hesitancy [25], limited research has used GT data to examine online interest in the HPV vaccine based on search behavior [4]. Information-seeking behavior is crucial to understanding public perception and readiness for health interventions. In an era of widespread internet use, GT serves as a valuable tool for tracking the public’s interest in specific health topics.

### Research Questions and Hypotheses

This study aimed to answer the following key research questions:

- What are the temporal trends in online information-seeking behavior about the HPV vaccine in India from 2010 to 2024?
- Are there specific inflection points in search interest that align with public health announcements, policy changes, or vaccine introductions?
- How can insights from online search behavior inform public health communication and strategies to promote HPV vaccine uptake?

The following hypotheses were made for this study:

- Public interest in the HPV vaccine, as reflected in Google search trends, would demonstrate significant increases corresponding to key public health events such as policy endorsements, vaccine introductions, and major communication campaigns.
- The increasing trend in internet penetration and digital engagement in India would lead to a consistent rise in HPV vaccine-related searches over the study period.

Therefore, this study aims to assess online information-seeking behavior in India regarding the HPV vaccine from January 2010 to April 2024, using GT data. The results of this infodemiological study will potentially inform strategies to promote HPV vaccine uptake.

## Methods

### Data Collection

We collected monthly search volumes and search queries for “HPV vaccine” from GT between January 1, 2010, and April 30, 2024; the GT data retrieval period was from May 1 to 15, 2024. GT provides a public database of the proportion of searches of a selected query performed on Google and presents the data as a relative search volume (RSV) in a normalized format. The data can be delineated by specific topics and search terms, time and year, and location. Specific to each search term, the RSV value ranges from 0 (minimal to no interest) to 100 (high popularity) based on the term’s search volume. An RSV value of 100 indicates the maximum search interest for the time and location selected relative to that specific term.

GT enables exploring online searches at different time intervals and retrieval queries for any keywords entered in the Google search engine. Using this technique, we retrieved monthly online search queries and normalized RSVs related to the HPV vaccine across states in India. GT allows for queries of both “search terms” and “search topics.” The “search terms” query provides the results for all keywords that fall within the category, and the “search topic” query renders the results of a group of terms that share the same concept in any language. We used both search terms and search topics to query results for “HPV vaccine.” The keywords “HPV Vaccine” and “Human Papillomavirus Vaccine” were used to capture RSVs for each year.

We used the framework described by Mavragani and Ochoa [26] for the region selection and period selection to retrieve query data from GT. Briefly, we searched for the keyword “HPV vaccine” at the country level (ie, India) to understand the overall RSVs in each year. All queries were searched between January 1, 2010, and April 30, 2024. The periods demonstrating high-value RSVs were further investigated by checking with news bulletins or the scientific literature to identify any events associated with these same periods.

### Statistical Analysis

This cross-sectional study analyzed online search trends for “HPV vaccine” in India over 14 years (January 2010 to April 2024) using GT. The annual mean RSV is used to summarize the online searches for each year between 2010 and 2024. The RSV metric is a normalized value representing the interest in a search term relative to the highest point of interest during the specified period. One-way analysis of variance followed by the Tukey post hoc test was performed to identify differences in HPV vaccine searches between Q1 to Q4 and between 2010 and 2024. A joinpoint regression analysis was performed for each year to analyze the time trend in the GT data using the Joinpoint Regression program (version 4.9.1.0) developed by the National Cancer Institute [27]. This software analyzes trends by regression modeling while searching for temporal trend changes at time points, called “joinpoints,” and estimates the regression function from previous joinpoints. This method allowed for the detection of inflection points in the data that corresponded with key public health announcements or events related to HPV vaccination, including policy changes and the launch of new vaccines. The number of joinpoints is obtained using a permutation test via Monte Carlo resampling, and the analysis criteria were set to find up to three joinpoints [28]. Data were retrieved for each year, with analysis focused on identifying trends in search behavior corresponding to major government initiatives and public health communication campaigns. Statistical significance was set at *P*<.05.

## Results

### Annual Trends in RSVs Related to HPV Vaccine

**Figure 1.**
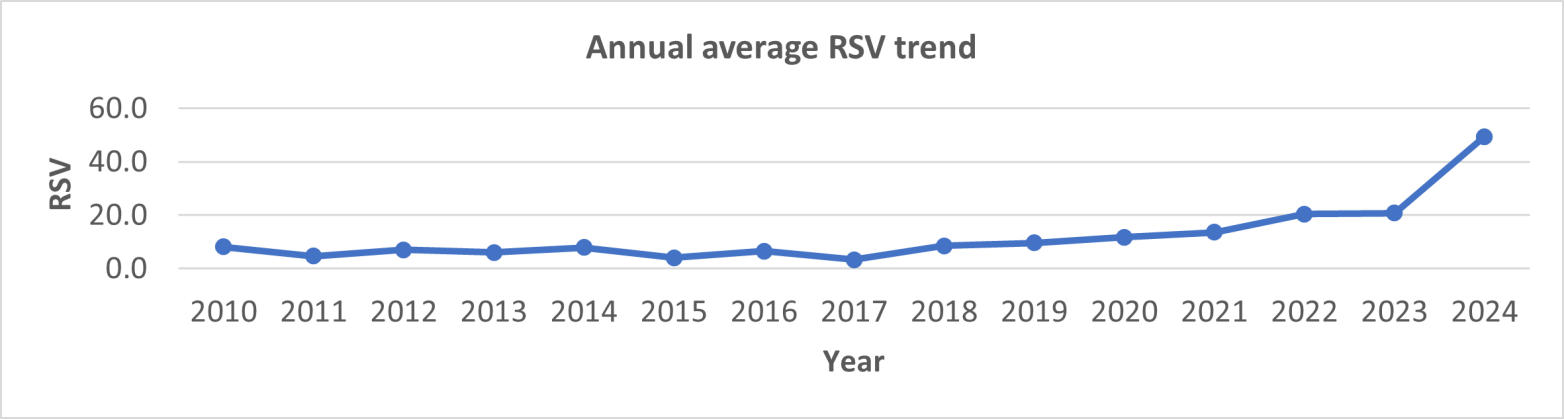
Annual average RSV trend for human papillomavirus vaccine from 2010 to 2024 obtained from Google Trends. RSV: relative search volume.

The study found that online search interest in the HPV vaccine increased remarkably over the study period, with an average annual growth of 13.7% (95% CI 7.9%‐19.1%) (Figure 1). The lowest average RSV was recorded in 2017 (3.38), and the highest in 2024 (49.5).

**Figure 2.**
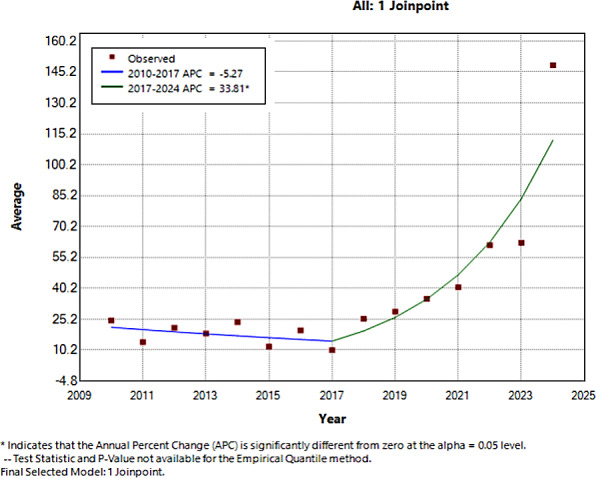
Joinpoint regression analysis of the annual percent change (APC*)*. The final selected model consisted of a single joinpoint.

The joinpoint regression analysis of the annual percent change is depicted in Figure 2. The final model selected consisted of a single joinpoint at 2017. A statistically significant rise in annual percent change is observed post 2017 (*P*<.05).

### Quarterly HPV Searches From 2010 to 2024

On analyzing the average quarterly HPV search volume from 2010 to 2024 (Table 1) using 1-way ANOVA, it was found that the F-ratio value is 0.39999 *(P*=.75). Therefore, the trend across different quarters of the year showed no statistically significant pattern.

**Table 1.** Quarterly differences in relative search volumes (RSVs) on Google Trends for the term “HPV vaccine” from 2010 to 2024 in India.

Year	2010, RSV	2011, RSV	2012, RSV	2013, RSV	2014, RSV	2015, RSV	2016, RSV	2017, RSV	2018, RSV	2019, RSV	2020, RSV	2021, RSV	2022, RSV	2023, RSV	2024, RSV
Q1	12.9	5.3	4.8	4.7	8.7	4.1	4.7	2.6	6.4	9.1	8.6	13	20.7	23	55.6
Q2	8.3	3.8	15.3	4.4	4.1	4.1	11.9	2.7	6.8	8.9	13.6	14.7	17.8	20.1	—^a^
Q3	7.6	5.1	4.1	11.3	4.7	4	7.3	2.3	10.8	12.7	11.1	15.6	24	19.2	—
Q4	4	4.6	3.9	4	14.3	3.8	2.6	6	9.9	7.9	13.6	11.2	19	20.8	—

a Not available.

Additionally, the post hoc Tukey test revealed that none of the pairs of quarters had a significant difference in the average RSV (Q1 and Q2: *P*=.99; Q1 and Q3: *P*=.98; Q1 and Q4: *P*=.99; Q2 and Q3: *P*=.99; Q2 and Q4: *P*=.98; Q3 and Q4: *P*=.97).

### Monthly Trends in HPV Vaccine Searches 2010-2024

**Figure 3.**
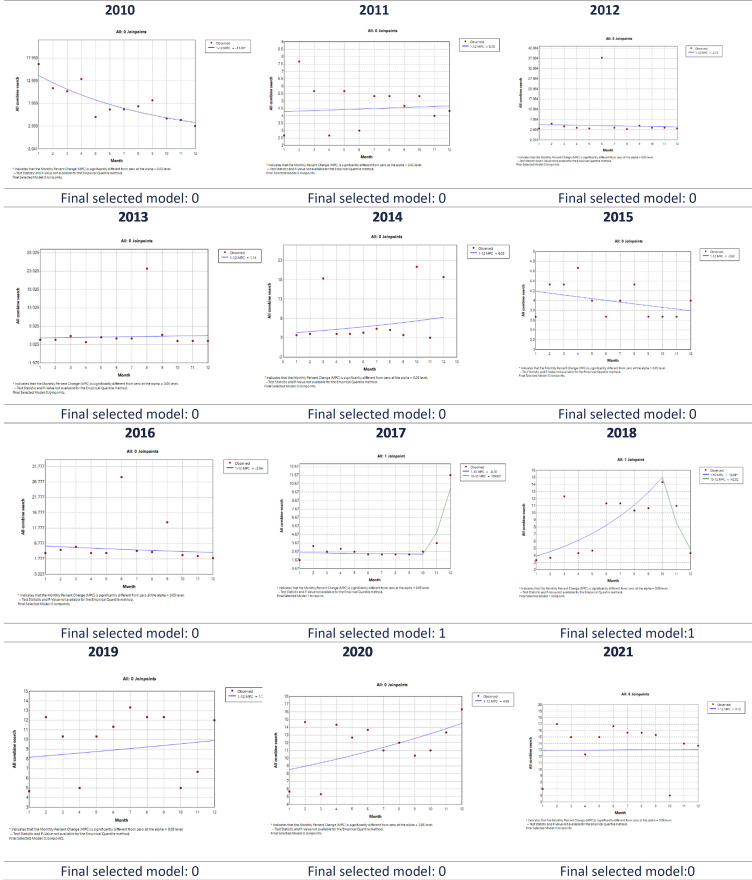
Joinpoint regression analysis indicating trends in “HPV vaccine” relative search volume on Google Trends from 2010 to 2024 in India. The number of slopes is determined by the number of joinpoints identified by the analysis. Joinpoints are the time points when statistically significant changes in the linear slopes are noted.

The joinpoint regression plots for each year from 2010 to 2024 are provided in Figure 3. Each graph provides corresponding monthly percent changes in the HPV vaccine searches in each full year. Only 2010 and 2017 showed a statistically significant trend when comparing monthly RSVs. While 2017 and 2018 reported a single joinpoint. All the other 13 years reported 0 joinpoints, suggesting no substantial change within a year.

## Discussion

The results of this study demonstrate a steady increase in the online information-seeking behavior among the public with respect to the HPV vaccine. Findings of the study show a steady and statistically significant drop in the RSV in 2010. This could be associated with the suspension of HPV vaccination trials in the country due to concerns regarding safety and ethical violations [29]. Additionally, a steep increase in the RSV post 2017 was observed. This substantial increase in public interest aligns with several key events, including the introduction of the HPV vaccine in select states of India [30]. In December 2017, the National Technical Advisory Group on Immunization recommended the inclusion of HPV in India’s Universal Immunization Programme [6]. Subsequently, in 2018, Sikkim State in India introduced a HPV vaccine for 9‐ to 13-year-old girls, primarily through school-based vaccination, targeting approximately 25,000 girls [31]. These landmark events in the sphere of the HPV vaccine were in line with the increase in online search inquiries.

In September 2022, the government of India announced India’s first indigenously developed vaccine, CERVAVAC, for the prevention of cervical cancer [32]. Following this, the Serum Institute of India launched CERVAVAC on January 24, 2023, by the hands of the then Hon’ble Home Minister [33]. These events saw a sustained search interest, indicating the public’s engagement with HPV-related health information.

Furthermore, a sustained search volume was seen in 2024 following announcements around initiatives planned for cervical cancer prevention in the interim budget. This search volume was maintained by a consistent presence in social media posts by community influencers and public figures. It must, however, be noted that when comparing the RSV with a previous study conducted in the United States, the RSVs are much lower for India [33]. This can be due to larger public interest in HPV vaccines in the United States due to a number of high impact activities occurring since 2010, such as the release of recommendations for the use of a quadrivalent HPV vaccine by the Centers for Disease Control and Prevention. Additionally, no trend or pattern was recorded when comparing the quarters of the year in terms of RSVs for the HPV vaccine. This was in contrast with the pattern observed in the United States, since a consistent pattern with a significant rise in RSV leading up to July and August followed by a drop in searches was found. This pattern of RSV peaks in July/August, aligning with the annual school calendar and the back-to-school period where HPV vaccination campaigns would be undertaken [34].

This research contributes to the expanding body of work exploring online and other digital data, and their application in health care and public health research. Specifically focusing on the HPV vaccine, we analyzed GT data to document online search patterns from 2010 to 2024. GT provides valuable insights into how public interest fluctuates in response to health policy announcements, vaccine launches, and public health campaigns. The spikes in search interest corresponding to public health events underscore the importance of timely and effective public health communication. Proactive engagement through digital platforms could play a critical role in addressing the information needs of the population, particularly in a country like India, where internet penetration is rapidly increasing. This study highlights the need for continued monitoring of online information-seeking behavior to inform public health strategies and interventions aimed at promoting HPV vaccination.

Despite our best efforts, this study has a few limitations. First, as GT data are observational, causal inferences (eg, an increase in HPV vaccine searches leading to higher vaccine coverage) cannot be made. However, we can leverage this infodemiology data, showing variations in online search trends over time and by topic, to customize health education and promotion materials related to HPV vaccination. These resources can be disseminated online and even used as targeted search engine advertisements during periods of high search activity. Second, our analysis was primarily conducted at the national level. While these data provide useful insights into overall online search behaviors and vaccine coverage, they do not capture relationships that may exist at more localized levels, such as within communities or states. Third, we are unable to pinpoint the exact causes behind fluctuations in search volume; we can only hypothesize potential associations based on known public health events and other relevant information. Additionally, the study end point involved only the health-seeking behavior of those with internet access, which is not representative of the entire population. However, understandably, it is impractical to assess all health-seeking practices. Thus, we assessed the more accessible, widely practiced, and subjective form of health-seeking behavior. Another potential limitation could be due to differential selection of search terms/input without inclusion of searches in any vernacular language, which could alter the RSVs. Furthermore, since we looked at Google search trends, the study design has selection bias due to the representation of only those individuals who had access to the internet. To overcome this bias to some extent, we have analyzed data from 2010 to 2024, as there has been a sharp increase in the use of the internet in recent times in India [35].

The results of this study provide valuable insights that can directly inform public health policy and future research. From a policy perspective, the identification of spikes in online search interest corresponding to significant events, such as vaccine endorsements and new vaccine introductions, suggests that public health campaigns should be strategically timed to coincide with these moments of heightened interest. Policy makers can leverage such trends to design tailored communication strategies that address public concerns, dispel misinformation, and reinforce the benefits of HPV vaccination. This could include using social media, local influencers, and vernacular content to effectively reach diverse populations. Furthermore, the study underscores the growing utility of digital tools like GT for monitoring public interest in health topics, providing policy makers with real-time insights to prioritize resources and interventions in regions or topics where search interest—and potentially awareness—may be low. These findings also highlight the importance of integrating digital data into broader public health strategies to dynamically assess the effectiveness of campaigns and adjust strategies accordingly.

For future research, the study opens avenues for more localized analyses, which could provide community-specific insights into barriers to HPV vaccine uptake, including cultural, linguistic, or socioeconomic factors. Additionally, research could explore the extent to which spikes in search interest translate into concrete actions, such as increased vaccine uptake, by integrating digital search data with vaccination records. There is also a need to further investigate the role of misinformation on search behaviors and its impact on vaccine hesitancy, with a focus on testing and evaluating proactive measures to counter misinformation with accurate and timely content. The framework used in this study has broader applicability and can be extended to other vaccines or public health interventions to monitor health information–seeking behaviors and guide strategies across various health topics.

This infodemiological study illustrates the value of GT in assessing public interest in health-related topics such as the HPV vaccine. To capitalize on periods of heightened online engagement, public health authorities should implement timely and targeted communication campaigns that coincide with key policy announcements or vaccine introductions. Digital health monitoring tools like GT should be integrated into routine vaccine awareness and promotion strategies, allowing for the early identification of emerging trends and real-time adjustments to outreach efforts. Additionally, localized, culturally relevant educational materials should be developed to address the diverse linguistic and socioeconomic landscape of India, ensuring that vaccine-related information reaches underserved populations. To combat misinformation, social media influencers, health care professionals, and trusted community figures should be mobilized to disseminate scientifically accurate content in engaging formats, such as short videos, infographics, and interactive question and answer sessions.

In conclusion, sustained public interest in HPV vaccination requires a proactive data-driven approach that leverages digital tools to inform public health strategies. By aligning communication efforts with public information-seeking behavior, policy makers and practitioners can enhance vaccine acceptance, counter misinformation, and foster long-term engagement with preventive health care. These insights not only apply to HPV vaccination but also set a precedent for using digital analytics to optimize other public health initiatives, ensuring that timely and relevant health information reaches the right audiences.

## Abbreviations:

GT: Google Trends
HPV: human papillomavirus
RSV: relative search volume
